# Non Linear Control System for Humanoid Robot to Perform Body Language Movements

**DOI:** 10.3390/s23010552

**Published:** 2023-01-03

**Authors:** Juan Manuel Gomez-Quispe, Gustavo Pérez-Zuñiga, Diego Arce, Fiorella Urbina, Sareli Gibaja, Renato Paredes, Francisco Cuellar

**Affiliations:** 1 Engineering Department, Pontificia Universidad Catolica del Peru, San Miguel, Lima 15088, Peru; 2 Department of Psychology, Pontificia Universidad Catolica del Peru, San Miguel, Lima 15088, Peru

**Keywords:** dynamic model, redundant manipulator, Backstepping control, Sliding Mode control

## Abstract

In social robotics, especially with regard to direct interactions between robots and humans, the robotic movements of the body, arms and head must make an adequate displacement to guarantee an adequate interaction, both from a functional and social point of view. To achieve this, the use of closed-loop control techniques that consider the complex nonlinear dynamics and disturbances inherent in these systems is required. In this paper, an implementation of a nonlinear controller for the tracking of trajectories and a profile of speeds that execute the movements of the arms and head of a humanoid robot based on the mathematical model is proposed. First, the design and implementation of the arms and head are initially presented, then the mathematical model via kinematic and dynamic analysis was performed. With the above, the design of nonlinear controllers such as nonlinear proportional derivative control with gravity compensation, Backstepping control, Sliding Mode control and the application of each of them to the robotic system are presented. A comparative analysis based on a frequency analysis, the efficiency in polynomial trajectories and the implementation requirements allowed selecting the non-linear Backstepping control technique to be implemented. Then, for the implementation, a centralized control architecture is considered, which uses a central microcontroller in the external loop and an internal microcontroller (as internal loop) for each of the actuators. With the above, the selected controller was validated through experiments performed in real time on the implemented humanoid robot, demonstrating proper path tracking of established trajectories for performing body language movements.

## 1. Introduction

Body language (i.e., gestures and bodily movements) is a key component of human communication [1,2]. Expressive bodily movements can convey information on their own [3,4] and constitute more than 50% of what we communicate to other people [5]. In fact, humans tend to be cued mainly by motion due to the emotional impact it has on them [4].

Current literature suggests that movements performed by robots are able to influence attitudes and perceptions toward them [2,6,7]. It has been found that robotic bodily expressions improve the understanding of affect (i.e., emotions and moods attributed to robots) [8], enhance the perception of trustworthiness [2,9], and awake empathetic responses toward robots [7]. Moreover, a combination of robotic speech and movements can increase feelings of familiarity [7] and foster human-like interaction [10]. Therefore, robotic movement becomes an important factor in interaction, both from a functional perspective and social perspective [7].

In the context of service robots there have been developments of common gestures that could be applicable in different social scenarios (e.g., companies, hospitals and schools) [11]. Examples of the most common gestures used during interactions with humans are: deictic gestures (e.g., pointing) to establish the identity or spatial location of an object, semaphoric gestures which are meant to send a specific predetermined message (e.g., waving), and gesticulation gestures that naturally accompanies common speech [12].

The mechatronic implementation of all these movements requires the use of automatic control techniques that consider the complex dynamics and disturbances inherent in these systems [13,14]. The automatic control system of humanoid robots typically consists of numerous interconnected computers and microcontrollers operating at multiple levels, involving low-level control of actuators for navigation and joint movements and high-level control for global displacement. This architecture implies different challenges in relation to system interconnection, event synchronization, related control loops, and fault diagnosis, among others [15,16,17]. Specifically, the use of closed-loop control methodologies that consider the non-linear dynamics of the system at its different operating points is required [17,18,19]. In this sense, the coordinated movements entail having to make use of controllers capable of understanding the dynamic of these system and sampling periods that can be variable [20]. In this sense, a reliable way to simulate and implement advanced controllers is the use of mathematical models, e.g., model-based predictive control, adaptative control, [21,22].

Specifically, in some studies [23,24], linear control proposals were presented and allow generating trajectories but with limitations in movements and speed. In other studies [25,26], the authors proposed slide mode control for robot path tracking, first for a quadrocopter and then for controlling the torque in each joint of the robot in order that the angle coordinates of each link coincide with the desired values. In [19], a Backstepping control proposal is presented for a robotic arm with satisfactory results in trajectory tracking obtained in simulation.

At Pontificia Universidad Católica del Perú (PUCP), the Qhali robot is under development. Qhali is an assistance robot designed to perform telepsychological interventions. Its design considered a humanoid appearance with two (02) articulated 4DOF arms and one (01) 2DOF head with an LDC display. Both elements allow the robot to perform gesture expressions with arms and head movements, aiming to improve the human–robot interaction during the interventions. The robot also has a navigation system that allows it to move automatically from one point to another. To communicate verbally and non-verbally, the robot has audio and video systems to achieve the ability to express emotions, gestures and body language whose purpose is to improve human–robot interaction [27]. The robot appearance and expression features were validated through a behavioral experiment to assess the perceived valence and meaning of the gestures performed.

In this article, the design and implementation of the arms and head of Qhali are initially presented, then the mathematical model of two arms with 4DOF and the head with 2DOF will be obtained by applying the physical principles. It is verified that with the increase in equations for the different necessary operating points, a highly non-linear model is obtained.

Then, the control design for this highly non-linear system is selected from the comparison of three non-linear controllers, such as non-linear PD control, Backstepping and Sliding Mode control, validating the results in simulation. Once the controller has been selected, the controller is implemented in ARM microcontrollers communicated with each of the servomotors through the CAN BUS protocol. Moreover, programming methods are presented which allow the use of these microcontrollers for centralized control in real time. In the implementation, first the validation of the obtained mathematical model is carried out and the control algorithm will be developed in the external loop that sends the necessary torque references to each motor where there will be an internal PI control loop.

Finally, the validations of the control strategies are performed from a generation of spline and polynomial trajectories. These experiments are analyzed in real time, obtaining all the information to MATLAB through a serial communication protocol to the PC. The experimental results allow verifying the effectiveness of a purely non-linear controller such as Backstepping compared to a partially adapted linear controller such as PD control with gravity compensation. The results are documented and discussed with which we will be able to obtain the conclusions of the work developed. Finally, the programming of these controllers and the generation of trajectories improve the movements performed by the arms and the head of the robot.

## 2. Design and Implementation of Arms and Head

The design of the arms and head of the humanoid robot was performed following the methodology VDI 2206 [28] used for the design of mechatronics systems. The aim of this design was to provide the robot with articulated arms and head to express a humanoid appearance. Moreover, by adding a head and arms the human–robot interaction could improve, as described in other studies [29].

### 2.1. System Design

As a first step of the methodology, the design requirements were defined as shown in Table 1. For this design, the torque required in each articulation was estimated through the static analysis of the extended position. The speed range was defined according to the approximate speed of human arms while performing regular activities. The angular speed of each joint was measured in [30]. Finally, the proportion of human dimensions was according to the anthropometric profile of Peruvian population in [31], which is necessary because the validation tests of the robot will be performed in Peruvian health centers.

**Figure 1:**
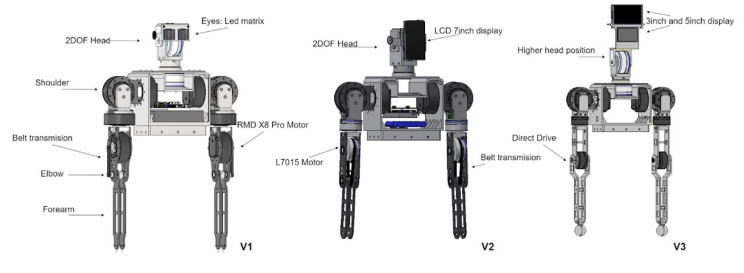
3D model of three conceptual designs of the robotic arms and head.

Following the design methodology, three (03) conceptual designs were developed and implemented to evaluate their performance. Figure 1 shows the three conceptual designs and their corresponding characteristics are as follows:

V1: four High torque servo motors, pulley and belt mechanism for 4th DOF, LED Matrix for eyes. V2: two High torque servo motors and two smaller, lower torque, pulley and belt mechanisms for 4th DOF, three small screens for eyes and mouth and V3: Joints directly driven by two high torque servo motors and two smaller, lower torque. Two screens for eyes and mouth.

**Figure 2:**
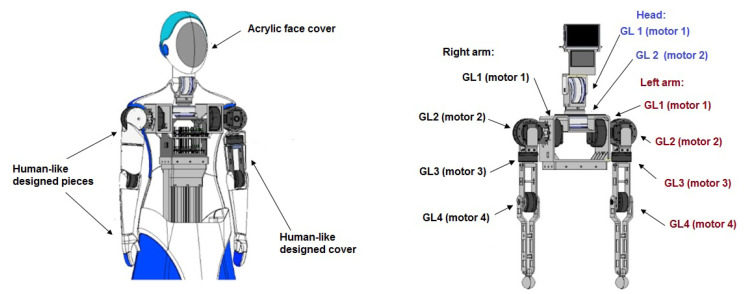
Visual representation of the design of humanoid and notation of each degree of freedom.

Based on a technical-economical evaluation, the third alternative was selected as the optimal conceptual design due to its performance and simplicity. Using this design, the upper body of the robot was included to the mobile robot platform, including the external structure. Figure 2 shows the visual representation of the robot and the notation of each degree of freedom (for each motor) used in this article for the arms and head.

### 2.2. Domain-Specific Design

**Figure 3:**
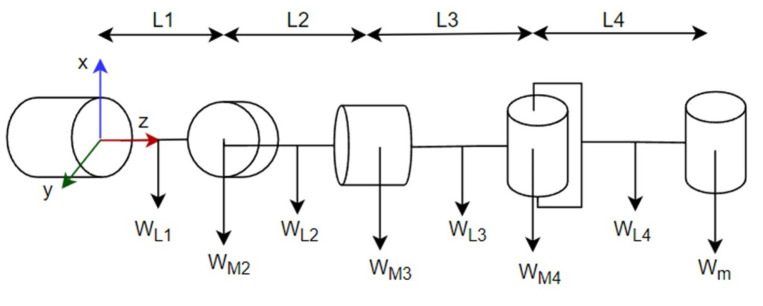
Free-body diagram of a horizontally extended arm.

The electronic and mechanical design was carried out. The actuators for the arms were selected according to the torque requirements of each joint. The maximum torque was calculated through a static analysis at the critical position of a horizontally extended arm, as shown in Figure 3 using Equations (1) and (2). In these equations, W corresponds to the weight of each element, L corresponds to the distance between actuators and T corresponds to the torque on each actuator. The maximum torque values obtained were 2.1 Nm for motors 1 and 2 and 0.36 for motors 3 and 4.

(1) $$\begin{array}{c} T_{4}=L_{4}\left(W_{m}+\frac{W_{L4}}{2}\right) \end{array}$$

(2) $$\begin{array}{c} T_{i}=T_{i+1}+L_{i}\left[W_{m}+\frac{W_{Li}}{2}+\left(\sum_{n=i+1}^{4}W_{Mn}+W_{Ln}\right)\right] \end{array}$$

**Figure 4:**
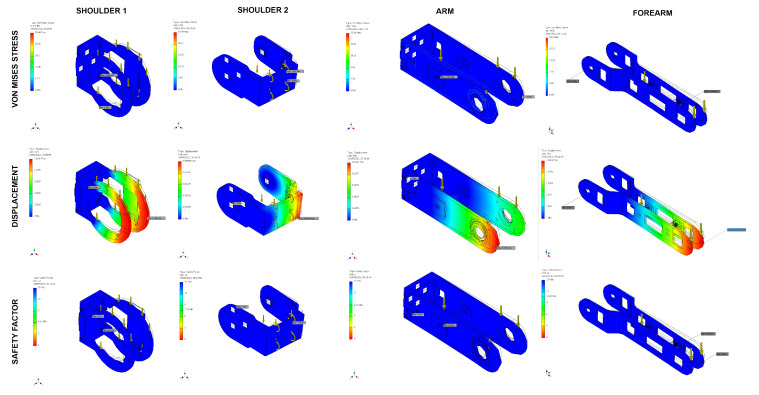
Finite element analysis on Autodesk Inventor of 3D printed arm pieces.

According to the requirements, the PLA 3D-printed structural pieces were designed to support the motor loads and to be as lightweight as possible, making each arm of a total weight of 2.31 kg. The critical elements, which required an structural analysis due to the forces generated with motion, are located in the arms. A total of four (04) different pieces were required in order to assemble the arms and head, as shown in Figure 4. Each piece resistance and deformation were verified through finite element analysis on Autodesk Inventor obtaining a maximum deformation of 0.18 mm and minimum security factor of 6.15, as shown in Table 2. This analysis confirms that the designed pieces will not fail during the implementation of the robot.

**Figure 5:**
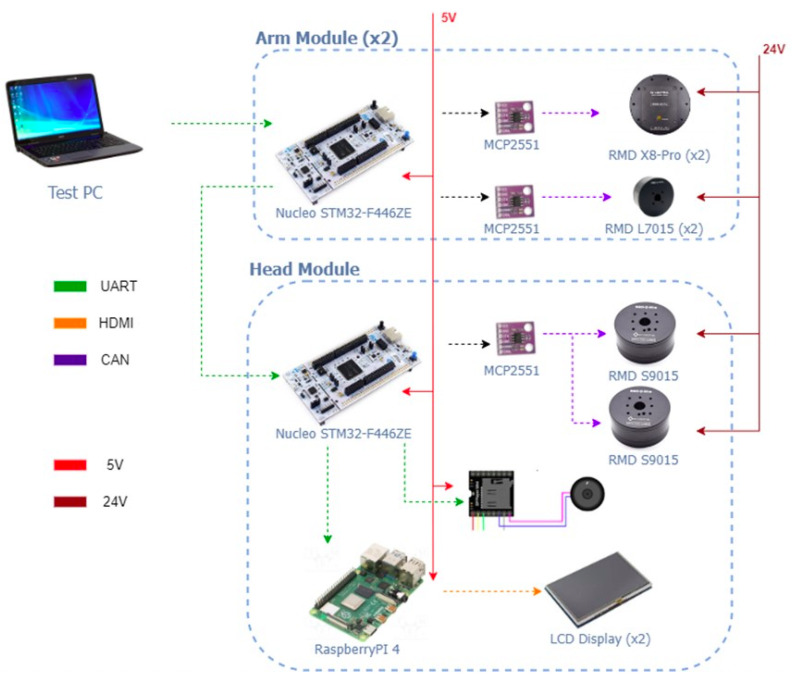
Hardware architecture of the robotic arms and head.

The chest piece, which was also 3D printed and contains the electronic boards to control and power the arms and head. It includes an audio subsystem (mp3 module and amplifier) and three (03) STM microcontrollers to interconnect different CAN buses and UART ports for communication with the actuators and other processors of the robot. The main chest piece also supports the head and the first arm joints, and connects the column with the upper body of the robot. The head has two (02) actuators, where their axes are perpendicular to allow 2DOF. The head has speakers on both sides, as well as two (02) screens of 5 and 3.5 inches, to emulate facial expressions, connected to a raspberry pi 4. Figure 5 represents the hardware architecture of the robotic arms and head.

### 2.3. System Integration

**Figure 6:**
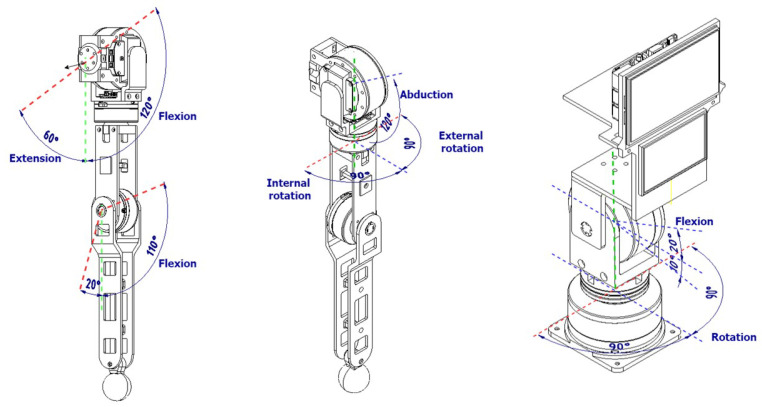
Movement capabilities of the integrated system.

**Figure 7:**
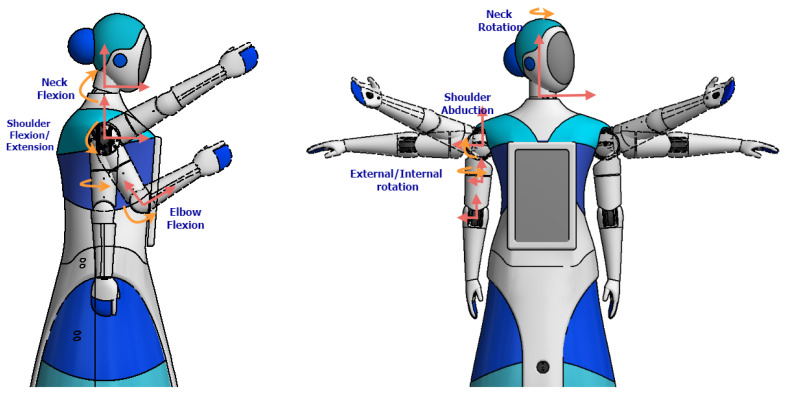
Movement of arms and head of the robot.

Based on the specific-domain design, a prototype was implemented to continue with the control design for the arms. The final design of the robot arms and head possess the capacities to perform movements such as the ones represented in Figure 6 and Figure 7. Moreover, Table 3 details additional technical information of the implemented prototype based on the design previously described.

## 3. Kinematic and Dynamic Analysis

The modeling of mechanical systems is obtained from the physical relationships that govern movement such as gravity, inertia, and effects such as Coriolis and friction. All these motion parameters are mathematically related in the Lagrange equation, which can become very complicated as the degrees of freedom of the system are larger than two. In Fu et al. [24], we observed a recursive procedure to obtain the parameters of this equation from the homogeneous transforms that are formed with the respective kinematic analysis. This recursive algorithm can be implemented, for example, in MATLAB to solve the summations and obtain the final equation offline, using the results to the algorithm in real time for the nonlinear controller.

### 3.1. Kinematic Analysis

**Figure 8:**
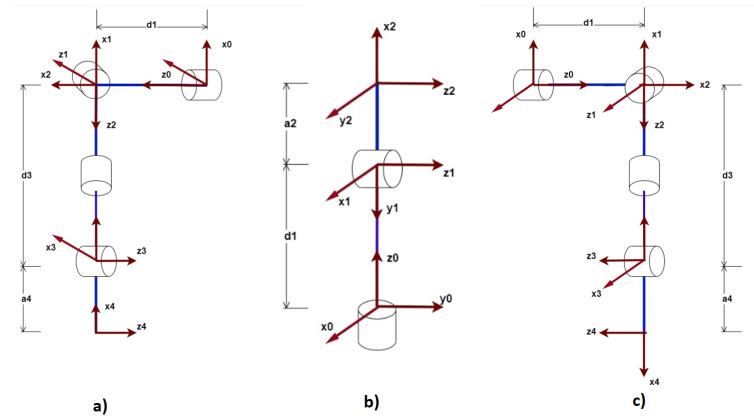
Denavit-Hartenberg analysis of arms and head (**a**) Right arm D-H analysis with 4 DOF; (**b**) Head D-H analysis with 2 DOF; (**c**) D-H analysis of left arm with 4 DOF.

From the design of the prototype presented in Section 2, it is possible to find the parameters of the system such as distances, masses and inertia. It is necessary, at this point, to perform the motion analysis without considering the forces that cause it. In this way, the Denavit-Hartenberg procedure [14] is applied as shown in Figure 8 for the arms and head of the robot.

When carrying out this analysis we obtain the homogeneous transform matrix arrays (4 × 4), that provide us with the information of the translation and rotation of a specific point of the system. In addition, this analysis will allow applying the inverse kinematics that allows the linking of an XYZ position in Cartesian space with its respective angular positions.

### 3.2. Dynamic Analysis

The application of the recursive algorithm explained in [24] gives us what is necessary to obtain the parameters of the robotic equation that will allow us to obtain the ideal torque $\tau_{\left(t\right)}$ at each instant of time according to the following expression:

(3) $$\tau =D_{\left(q_{t}\right)}{\ddot{q}}_{\left(t\right)}+h_{\left(q_{\left(t\right)},{\dot{q}}_{\left(t\right)}\right)}+c_{\left(q_{t}\right)}+Df,$$

where $D_{\left(q_{\left(t\right)}\right)}$ is a 4 × 4 matrix called inertia matrix, $h_{\left(q_{\left(t\right)},{\dot{q}}_{\left(t\right)}\right)}$ and $c_{\left(q_{\left(t\right)}\right)}$ are 4 × 1 matrixes called Coriolis matrix and gravity, respectively, and q(t) is the 4 × 1 matrix that contains the angular positions of the system, see the detail in the following expressions [24]:

(4) $$D=\begin{array}{ccc} \left[\begin{array}{cccc} d_{11} & d_{12} & d_{13} & d_{14}\\ d_{21} & d_{22} & d_{23} & d_{24}\\ d_{31} & d_{32} & d_{33} & d_{34}\\ d_{41} & d_{42} & d_{43} & d_{44} \end{array}\right] & H=\left[\begin{array}{c} h_{12}\\ h_{22}\\ h_{23}\\ h_{42} \end{array}\right] & C=\left[\begin{array}{c} c_{12}\\ c_{22}\\ c_{23}\\ c_{42} \end{array}\right] \end{array}$$

On the other hand, $D_{f}$ is a 4 × 1 matrix that represents the frictions that occur in the system and that better resemble the mathematical model to the real system. There are various mathematical models to represent these frictions, one of these models is represented by the following expression:

(5) $$D_{f}=f_{v}\dot{q}+f_{c}sign\left(\dot{q}\right)$$

where $\dot{q}$ is a 4 × 1 matrix representing the angular velocity, $f_{v}$ and $f_{c}$ are 4 × 4 diagonal matrixes with elements to be found from experimental tests. For the simulation of the designed controller, the values used for friction were those that best emulate the behavior of robotic systems. Later, these values will be found experimentally.

**Figure 9:**
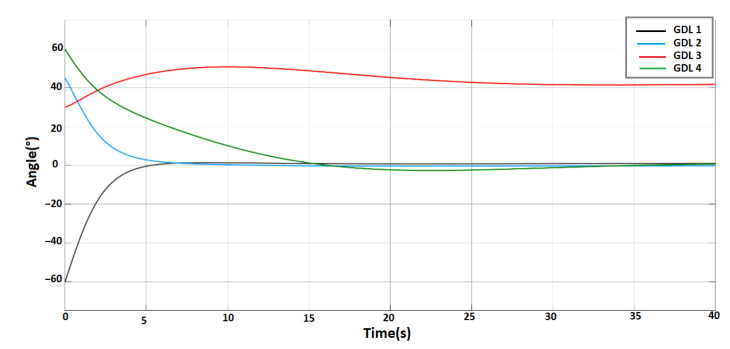
System response to different initial conditions: −60${}^{{}^{\circ}}$, 45${}^{{}^{\circ}}$, 30${}^{{}^{\circ}}$, 60${}^{{}^{\circ}}$.

After obtaining all the system parameters of the robotic equation, several simulated tests have been performed to verify and validate the mathematical model found. In Figure 9, we can observe the response of the system for an initial condition different from the ZERO position of the arms.

This is how we can verify that the system obtained is stable. The procedure is similar for both arms. In this way, we obtain the mathematical models of the arms and even for the head, considering the latter as a 2 × 2 matrix system. These models will be used to design and implement the control system.

## 4. Non-Linear Control Design

**Figure 10:**
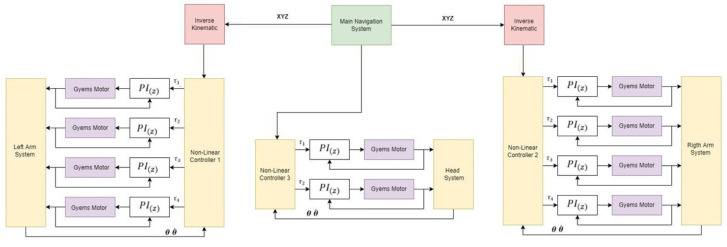
Proposed control scheme for the movements of the teleoperated robot.

The control architecture of the system is composed of a main controller in the external loop that has as references the position θ and the speed $\dot{\theta }$ of each of the motors in vector arrays. The output of the controller will be the necessary torque τ that each motor will have to provide and they will have an internal torque regulation loop through a PI control and in this way guarantee that the desired results are obtained. Figure 10 shows the general scheme of the controllers to be designed for each extremity of the robot.

In the case of internal PI control for each motor, the controller provided by the motor through its own software will be used, to which tuning tests will be applied to establish the most appropriate proportional and integrative parameters for each degree of freedom. In the case of the external loop, there is a nonlinear control that has been selected based on simulation tests in MATLAB according to the mathematical model obtained. Below is the comparison of the controllers.

### 4.1. PD Control with Gravity Compensation (PD+G)

It is known that PID controllers are quite simple in their application to various systems since prior knowledge of the system is not required. In the case of robotic systems, applying an integrator could cause the control to limit the system to slower movements than required. A solution to this problem is to use a gravity compensator that provides the system with a variable bias in each move to obtain zero stationary error. The gravity compensator is added to the previous PD control law and forms a non-linear controller. It is possible to demonstrate the convergence of this controller if we apply the controller that has the following form:

(6) $$\tau =K_{p}\tilde{q}-K_{d}\dot{q}+G\left(q\right)$$

where $K_{p}$ and $K_{d}>0\in R^{nxn}$ are diagonal matrixes of proportional and derivative gains, respectively. Furthermore, the position error $\tilde{q}$ is defined as follows:

(7) $$\tilde{q}=q_{d}-q$$

Then replacing Equation (6) in the robotic equation, the following system is obtained:

(8) $$D_{q(t)}\ddot{q}\left(t\right)+h_{\left(q\left(t\right),\dot{q}\left(t\right)\right)}=K_{p}\tilde{q}-K_{d}\dot{q}$$

which can be represented in matrix form by:

(9) $$\frac{d}{dt}\left[\begin{array}{c} \tilde{q}\\ \dot{q} \end{array}\right]=\left[\begin{array}{c} -\dot{q}\\ D_{q(t)}^{-1}\left(K_{p}\tilde{q}-K_{d}\dot{q}-h_{\left(q\left(t\right),\dot{q}\left(t\right)\right)}\right) \end{array}\right]$$

Considering the following expression as a Lyapunov function:

(10) $$V\left(\tilde{q},\dot{q}\right)=\frac{1}{2}{\dot{q}}^{T}D_{q(t)}\dot{q}+\frac{1}{2}{\tilde{q}}^{T}K_{p}\tilde{q}$$

Its time derivative:

(11) $$\dot{V}\left(\tilde{q},\dot{q}\right)={\dot{q}}^{T}D_{q(t)}\ddot{q}+\frac{1}{2}{\dot{q}}^{T}{\dot{D}}_{q(t)}\dot{q}+{\tilde{q}}^{T}K_{p}\dot{\tilde{q}}$$

Furthermore, considering the properties of the matrixes of the robotic equation, the following result can be reached:

(12) $$\dot{V}\left(\tilde{q},\dot{q}\right)=-{\dot{q}}^{T}K_{d}\dot{q}\leq 0$$

With which it is verified that the origin is stable and using the Lasalle invariance principle, the global asymptotic stability can be demonstrated using the following omega function:

(13) $$\Omega =\left\{\left[{\tilde{q}}^{T},{\dot{q}}^{T}\right]\in R^{2n}:\dot{V}\left(\tilde{q},\dot{q}\right)=0\right\}$$

Since for Ω it is true that $\dot{V}\left(\tilde{q},\dot{q}\right)=0$ if and only if $\dot{q}=0$ then $\ddot{q}=0$. From Equation (9) it follows that:

(14) $$0=D_{q(t)}K_{p}\tilde{q}$$

Then $\tilde{q}=0$ which ensures that $\dot{V}\left(\tilde{q},\dot{q}\right)<0$ for all ${\left[{\tilde{q}}^{T},{\dot{q}}^{T}\right]}^{T}\neq 0$ and in this way the global system is asymptotically stable.

Similarly, [14] shows methods for tuning this controller. One way is to do it by obtaining the mathematical model gravity matrix, from which the maximum eigenvalues of its gradient matrix are obtained. In this way, the following profit values have been chosen:

(15) $$\begin{array}{cccc} K_{p}=diag\{5.4781 & 13.3128 & 2.226 & 21.3205\}\\ K_{d}=diag\{3.31 & 5.16 & 2.11 & 6.53\} \end{array}$$

### 4.2. Backstepping Control (BC)

It is a controller based on the mathematical model of the system shown in Equation (3). Its main disadvantage is repeating the differentiation of virtual inputs, which increases the complexity of the controller [32]. The literature presents as solutions dynamic surface control based on the fractional order filter [33] and disturbance observer [34]. Another drawback of BC is that the system must be written in the strict feedback form [35], for which many solutions have been represented in the literature for avoiding this disadvantage such as a model-free back-stepping normal form and block back-stepping [35]. Some solutions developed are robust adaptive back-stepping control [36] and radial basis function neural network [37].

The efficiency of this controller lies mainly in the choice of a successful system model. This controller, as in other nonlinear controllers, bases its design on the stability and convergence criteria of the closed-loop system from Lyapunov functions. The procedure to find the control law starts from the remodeling of the robotic Equation (3) to a state space system of vector form as follows:

(16) $$\begin{array}{cc} & \left\{\begin{array}{c} x_{1}=q\\ x_{2}={\dot{x}}_{1}=\dot{q}\\ {\dot{x}}_{2}=\ddot{q}={D_{\left(q_{\left(t\right)}\right)}}^{-1}\cdot \left(\tau -h_{\left(q_{\left(t\right)},{\dot{q}}_{\left(t\right)}\right)}-c_{\left(q_{\left(t\right)}\right)}-Df\right) \end{array}\right. \end{array}$$

Then the state space equations would be:

(17) $$\begin{array}{cc} & \left\{\begin{array}{c} {\dot{x}}_{1}=x_{2}\\ {\dot{x}}_{2}={D_{\left(q_{\left(t\right)}\right)}}^{-1}\cdot \left(\tau -h_{\left(q_{\left(t\right)},{\dot{q}}_{\left(t\right)}\right)}-c_{\left(q_{\left(t\right)}\right)}-Df\right)=w \end{array}\right. \end{array}$$

Please note that the variable *w* has been used to represent the entire expression of ${\dot{x}}_{2}$ and considering the virtual control variable $x_{2}=v$, is obtained:

(18) $${\dot{x}}_{1}=v$$

For this system, the first Lyapunov function and its following derivative are considered:

(19) $$\begin{array}{ccccc} V_{1}=\frac{1}{2}x_{1}^{2}; & V_{1}\left(0\right)=0; & V_{1}\left(x\right)>0 & x\neq 0; & {\dot{V}}_{1}=x_{1}{\dot{x}}_{1} \end{array}$$

To show that ${\dot{V}}_{1}<0$ we must take $v=-K_{1}x_{1}$ where $K_{1}>0$ and replacing we obtain:

(20) $$\begin{array}{cc} {\dot{V}}_{1}=-K_{1}x_{1}^{2} & K_{1}>0 \end{array}$$

Then the stability of the system must be demonstrated to ensure that our virtual variable complies with what is required. This new system is given by:

(21) $$\begin{array}{cc} z=x_{2}-v & \lim_{x\to \infty }\left(z\right)=0 \end{array}$$

From Equation (21) the following is obtained:

(22) $$\begin{array}{ccc} z=x_{2}+K_{1}x_{1} & ⟹ & x_{2}={\dot{x}}_{1}=z-K_{1}x_{1} \end{array}$$

By deriving Equation (22) and considering the original system of Equation (17) and knowing that ${\dot{x}}_{1}=x_{2}$, then results:

(23) $$\begin{array}{ccc} z=x_{2}+K_{1}{\dot{x}}_{1} & ⟹ & \dot{z}=w+K_{1}x_{2} \end{array}$$

By replacing Equation (22) in Equation (23), the final expression of the system to be analyzed is obtained:

(24) $$\dot{z}=w+K_{1}\left(z-K_{1}x_{1}\right)$$

To achieve the stability of this system, the following Lyapunov function and its derivative are considered:

(25) $$\begin{array}{cccc} V=V_{1}+\frac{1}{2}z^{2}=\frac{1}{2}x_{1}^{2}+\frac{1}{2}z^{2}; & V(x)>0 & x\neq 0; & \dot{V}=z\dot{z}+x_{1}{\dot{x}}_{1} \end{array}$$

To show that $\dot{V}<0$, the results of Equations (23) and (24) are replaced in Equation (25) obtaining

(26) $$\dot{V}=x_{1}\left(z-K_{1}x_{1}\right)+z\left(w+K_{1}\left(z-K_{1}x_{1}\right)\right)$$

Rearranging the last expression:

(27) $$\dot{V}=-K_{1}x_{1}^{2}+z\left(w+K_{1}\left(z-K_{1}x_{1}\right)+x_{1}\right)$$

It is evident that to obtain that $\dot{V}<0$ it is only necessary to do the following:

(28) $$\begin{array}{cc} w+K_{1}\left(z-K_{1}x_{1}\right)+x_{1}=-K_{2}z & K_{2}>0 \end{array}$$

In this way, it is obtained:

(29) $$\dot{V}=-K_{1}x_{1}^{2}-K_{2}z^{2}$$

Thus, this last result allows us to prove that $\dot{V}<0$ and obtain that the system will be globally asymptotically stable.

From Equation (27), replacing *w* with its original expression given in Equation (17) and solving

(30) $$D_{\left(q(t)\right)}^{-1}\left(\tau -h_{\left(q_{\left(t\right)},{\dot{q}}_{\left(t\right)}\right)}-c_{\left(q_{\left(t\right)}\right)}-Df\right)=-\left(K_{1}+K_{2}\right)z+\left(K_{1}^{2}-1\right)x_{1}$$

From equation Equation (22), *z* is replaced and rearranging the expression

(31) $$D_{\left(q(t)\right)}^{-1}\left(\tau -h_{\left(q_{\left(t\right)},{\dot{q}}_{\left(t\right)}\right)}-c_{\left(q_{\left(t\right)}\right)}-Df\right)=-\left(K_{1}K_{2}+1\right)x_{1}-\left(K_{1}+K_{2}\right)x_{2}$$

Then returning to the initial variables $x_{1}=q$; $x_{2}=\dot{q}$ and isolating τ from the last expression:

(32) $$\tau =h_{\left(q_{\left(t\right)},{\dot{q}}_{\left(t\right)}\right)}+c_{\left(q_{\left(t\right)}\right)}+Df-D_{\left(q(t)\right)}\left(K_{1}K_{2}+1\right)q-D_{\left(q(t)\right)}\left(K_{1}+K_{2}\right)\dot{q}$$

Since it has been shown that the system is globally asymptotically stable, it can be obtained for a desired $q^{*}$ and ${\dot{q}}^{*}$, the final expression of the control signal, $q^{*}$ and ${\dot{q}}^{*}$ are the positions and angular velocities to be reached, respectively.

(33) $$\tau =h_{\left(q_{\left(t\right)},{\dot{q}}_{\left(t\right)}\right)}+c_{\left(q(t)\right)}+Df-D_{\left(q(t)\right)}\left(K_{1}K_{2}+1\right)\left(q-q^{*}\right)-D_{\left(q(t)\right)}\left(K_{1}+K_{2}\right)\left(\dot{q}-{\dot{q}}^{*}\right)$$

This is how the Backstepping control signal to be applied to the system is obtained. This expression depends on the knowledge of the expression assigned to the mathematical model of the robotic model. Furthermore, the only condition for the gains $K_{1}$,$K_{2}$ is that both are positive matrixes, so we have initially considered the following gains:

(34) $$\begin{array}{cccc} K_{p}=diag\{10 & 10 & 10 & 10\}\\ K_{d}=diag\{10 & 10 & 5 & 5\} \end{array}$$

During the experimental tests, it is observed that as the values of these gains increase, a faster stable system is still obtained, but the necessary τ will be greater, thus a limitation, during the implementation, for these gains will be the maximum capacity of each actuator to use.

With this procedure, the necessary torques are obtained so that the system can reach the requested points with the required speeds. An important advantage that could be observed is that the control law does not require the inverse matrix operator, which will be a great advantage when implementing it.

### 4.3. Sliding Mode Control (SMC)

The main objective of this nonlinear control is to position the system to the desired operating point using a sliding region. Once the operating point is reached, the control variable will initiate oscillations, known as Chattering, to maintain the operating point. These discontinuous changes are harmful to the actuator, to avoid this damage there are several methods.

The literature presents several solutions to overcome the chattering effect, such as the global high sliding mode controller with a continuous component [38], adaptation mechanism [39], extended state observer [40], chatter-free twofold sliding mode control [41], fuzzy logic [42] and saturation function [43].

In [26], a way to arrive at the SMC control law is shown from the analysis of a system of order “n”. In this work, this analysis is used for its application in a second order system that is the mathematical model found. This second order system can be represented by the following expression:

(35) $$\ddot{x}=f\left(x,t\right)+u\left(t\right)$$

Considering that the error is defined as follows:

(36) $$e=x_{set}-x$$

where $x_{set}$ is the desired operating point, the following sliding region and its derivative are defined:

(37) $$\begin{array}{cc} s=\dot{e}+\lambda e; & \dot{s}=\ddot{e}+\lambda \dot{e} \end{array}$$

By replacing Equation (36) in Equation (37), the following expression is obtained:

(38) $$\begin{array}{c} \dot{s}={\ddot{x}}_{set}-\ddot{x}+\lambda \left({\dot{x}}_{set}-\dot{x}\right)\\ \dot{s}={\ddot{x}}_{set}-f\left(x,t\right)-u\left(t\right)+\lambda \left({\dot{x}}_{set}-\dot{x}\right) \end{array}$$

To demonstrate the stability of the system, the following Lyapunov function and its derivative are used:

(39) $$\begin{array}{cccc} V=\frac{1}{2}s^{2}; & V(x)>0 & x\neq 0; & \dot{V}=s\dot{s} \end{array}$$

Based on the proposal of [26] where we select the following

(40) $$\begin{array}{cc} \dot{s}=-Ksign\left(s\right) & K>0 \end{array}$$

To obtain the following expression,

(41) $$\dot{V}=s\left(-Ksign\left(s\right)\right)=-K\left(s\right)<0$$

This shows that the system is globally asymptotically stable. Now, obtaining Equation (39) is only possible if u(t) is equal to the following:

(42) $$u\left(t\right)=-f\left(x,t\right)+{\ddot{x}}_{set}+\lambda e+Ksign\left(s\right)$$

We obtain in this way the control law to use. It must be considered that the application of this last result must consider that the system is of dimension 4 × 1 and also as follows:

(43) $$\begin{array}{cc} u(t)=\tau ; & \mathrm{Applied}\mathrm{torque}\\ f\left(x,t\right)=D_{\left(q(t)\right)}\ddot{q}\left(t\right)+h_{\left(q\left(t\right),\dot{q}\left(t\right)\right)}+c_{\left(q(t)\right)}+D_{f}; & \mathrm{Mathematical}\mathrm{model}\\ {\ddot{x}}_{set}={\ddot{q}}^{*}; & \mathrm{Desired}\mathrm{acceleration}\\ e=q^{*}-q; & \mathrm{Error} \end{array}$$

where λ and *K* are diagonal matrixes of dimension 4 × 4. Thus, the final control law is as follows:

(44) $$\tau =-D_{\left(q(t)\right)}\ddot{q}\left(t\right)-h_{\left(q\left(t\right),\dot{q}\left(t\right)\right)}-c_{\left(q(t)\right)}-D_{f}+{\ddot{q}}^{*}+\lambda e+Ksign\left(s\right)$$

The direct application of this control law produces chattering.

**Figure 11:**
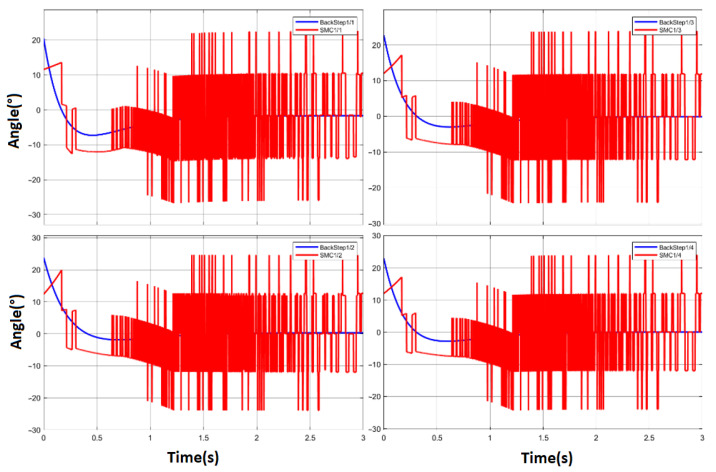
Chattering caused by the SMC control without saturation at 4 DOF of the robotic manipulator.

At this point, having both the system model and the SMC controller, a first simulation of the system is performed where saturation is not applied. In Figure 11, we can see the chattering produced by the controller without the use of saturation.

To avoid this condition, in [26] the change of the term Ksign(s) of Equation (44) is proposed for the following saturation condition:

(45) $$\begin{array}{cc} {\dot{s}}_{1}=K_{i}sat\left(s_{i}\right)= & \left\{\begin{array}{c} \begin{array}{ccc} K_{i}sign\left(s_{i}\right) & if & |s_{i}|>0\\ K_{i}\frac{s_{i}}{d} & if & |s_{i}|\leq d \end{array} \end{array}\right. \end{array}$$

In this way, the parameters to be used in the SMC control are λ, *K* and delay, where λ directly influences the speed with which the system reaches the desired operating point, *K* will be the allowed gain that the actuator will use to maintain the operating point and delay is the parameter that will avoid the discontinuities of the sign function. The initial values for these parameters will be the following:

(46) $$\begin{array}{cccc} \lambda =diag\{3 & 3 & 3 & 3\}\\ K=diag\{15 & 15 & 15 & 15\}\\ delay=diag\{1 & 1 & 1 & 0.1\} \end{array}$$

Let us remember that as the saturation is greater, indeed, the oscillations of the actuator will be less significant, but the SMC control will be more sensitive to disturbances.

### 4.4. Comparison of the Proposed Controllers

With the three (03) controllers designed and implemented using the MATLAB simulation software, it is possible to perform the tests that facilitate the comparison and the necessary conclusions for the choice of the controller that will be implemented in hardware.

**Figure 12:**
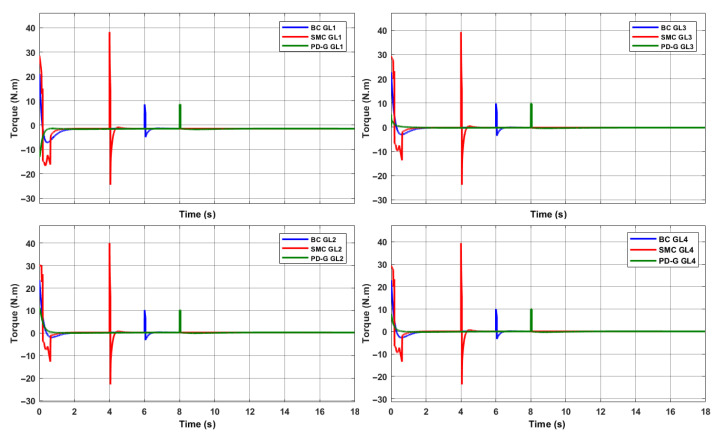
Torques applied with the controllers proposed to the 4DDL of the manipulator.

The first test is the comparison of the responses of each controller to a step input (SP: −75${}^{{}^{\circ}}$, 40${}^{{}^{\circ}}$, 55${}^{{}^{\circ}}$, 90${}^{{}^{\circ}}$), then a steady-state torque disturbance has been added for each controller as shown in Figure 12.

**Figure 13:**
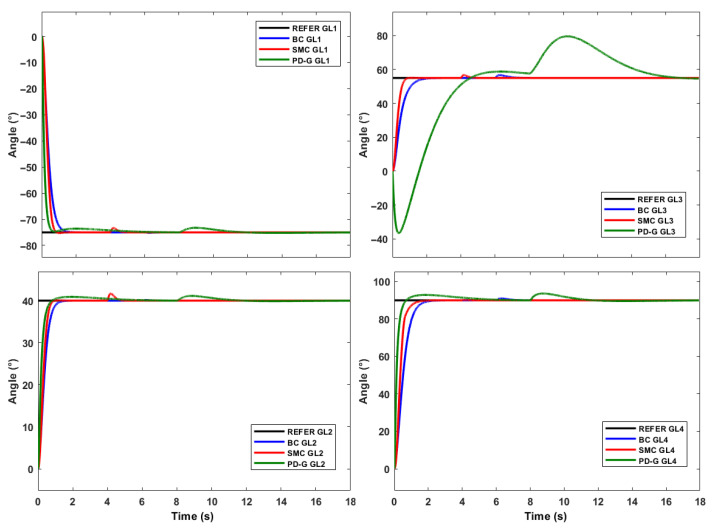
Positions of 4 manipulators applying a torque disturbance.

A disturbance is observed of the same magnitude that has been applied at different times for each controller, with the SMC control being the one that requires the highest torque value to adequately regulate the requested position of each servomotor. Figure 13 shows the positions generated with this control variable applying a torque disturbance for SMC in 4 s, BC in 6 s and PD+G in 8 s.

We can appreciate that the regulation is faster for the BC and SMC, with the advantage that the Backstepping requests smoother torque changes than the Sliding Mode control. On the other hand, we see that the SMC is much faster and more robust to disturbances than the BC.

**Figure 14:**
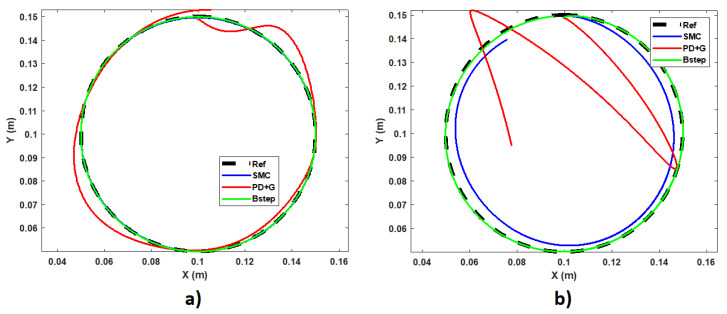
Cartesian motion of the end effector at different freq. (**a**) w = 0.2 rad/s, (**b**) w = 2.1 rad/s.

A second test performed on the proposed controllers is the application of a variable frequency input. For this case, the generation of trajectories have been developed through the parametric curve of the circumference. In this way we will be able to observe some advantages and deficiencies of each controller. In Figure 14, we can observe the movement with respect to a circumference.

**Figure 15:**
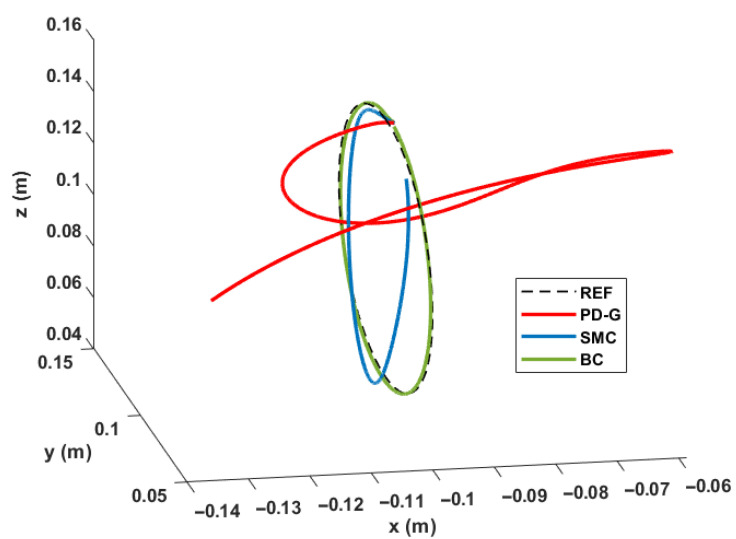
Comparison of controllers for the 3D movement of the end effector to w = 2.1 rad/s.

In the first case, the circle can be completed in a time of 32 s; while, in the second case, the execution time is 3 s. In this way, we can see that the slower the movement, all the controllers can respond adequately to the tracking of trajectories, but it is at higher speeds where the effectiveness of purely non-linear controllers such as SMC and BC can be observed. In Figure 15, we can see this same comparison in a 3D plane at the angular frequency of 2.1 rad/s.

It is important to mention that, for the purposes of a better visualization of the simulations presented, it has been started at a point on the circumference and in this way avoid the initial position error that is easily overcome by SMC and BC, but not by the PD+G controller. At the end of the simulation tests, we can observe that the three controllers can achieve the requested requirements, but it is the PD controller with gravity compensation that could not be used due to its sensitivity to disturbances and its ineffectiveness when needed perform faster movements. Let us remember that the main application of the arm is not governed by the accuracy with which the movements are made, but by the degree of nature to which the movements are made, which in many cases is mostly determined by speed of motions.

A test to compare the efficiency of the proposed controllers can be performed from the generation of trajectories using third or fifth order polynomials [14], with which better position and velocity profiles can be provided to the movements to be performed. This generation of trajectories from polynomials will allow us to define routes for the movement of the arms, for which different Cartesian points are defined that the end effector of the arms must reach.

Table 4 summarizes the results found for the comparison of the three controllers based on the simulation results of the implementation of these polynomial trajectories.

As can observed in Table 4, the SMC controller has the best regulation and monitoring in closed-loop control. However, the comparison considers factors that may be decisive for the implementation of the algorithm in the hardware. Consequently, the BC is selected, which has results very close to the SMC controller and provides some important advantages for the implementation such as execution time and smoothness of actuator changes.

Thus, for the present work, the use of BC was agreed as the main controller for the movements to be performed by the arms and head of the robot.

## 5. Implementation

The arms and head of the robot are composed of an ARM microcontroller on a central STM32 development board for each limb and built-in GYEMS motors with CAN BUS protocol and a PI electronic controller for the torque to be regulated. In addition, each motor has a built-in encoder that provides the position and speed in real time, which allows obtaining the proposed control loops.

**Figure 16:**
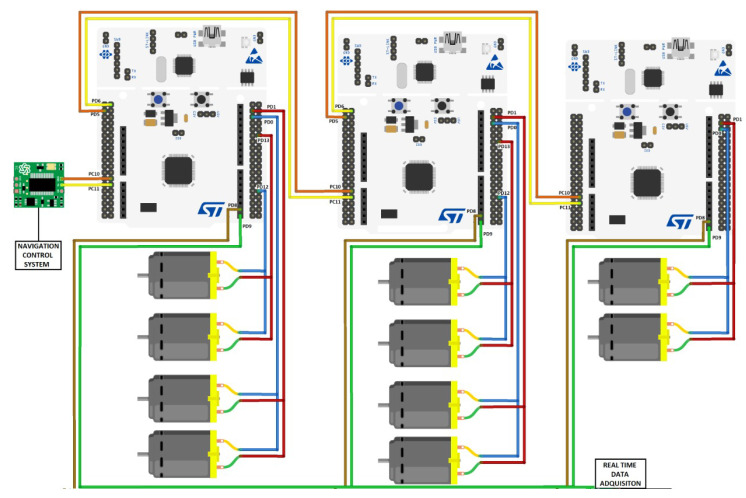
Connection diagram for the system control.

In the case of the STM32, it is an embedded 512 Kbyte 32-bit ARM Cortex-M microcontroller with two (02) CAN BUS ports for communication with the motors at each end of the robot and serial UART port for communication between all the ends of the ROBOT and the transmitted data to an analyzer such as MATLAB. To optimize the communication times of the CAN BUS protocol, it has been decided to use the two (02) CAN BUS ports of each STM32 card, connecting up to two (02) motors in each port. In this way, it has been possible to achieve a communication time of less than 20 ms. This time was used as the sampling time for the closed-loop controls. Figure 16 shows a scheme of the connections implemented.

The STM32 cards share a serial bus one after the other through the UART protocol where they receive the selection of the actions to be performed by the limbs from the central navigation system. An additional UART port has also been implemented in each STM32 for the output of data in real time that will be used for the presentation of the results obtained in the experiments performed using the MATLAB software.

### 5.1. Validation of the Mathematical Model

The next step in the implementation is to verify that the mathematical model of the system is correct. As mentioned, most of the model parameters have been obtained from the CAD design of the worked prototype. However, only heuristic values were used for the proposed friction model. In [44], an experimental method is presented to find all the coefficients related to frictions. The procedure starts from a PD controller with gravity compensation which will maintain a constant speed in the motors for a window of time where the average speed and torque can be obtained. This experiment is performed several times until a speed and torque map is obtained which, based on linear regressions, will allow us to obtain the required coefficients.

**Figure 17:**
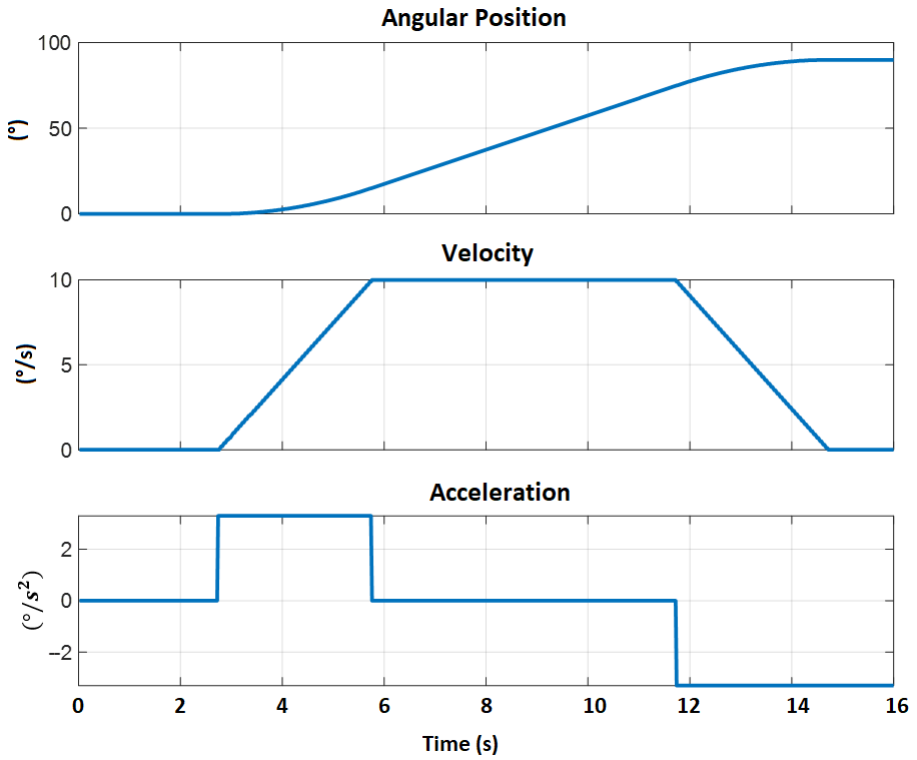
Speed profile applied to each motor from the STM32 card.

In this case, it was necessary to implement the PD controller with gravity compensation applied to a generation of trajectories with a trapezoidal profile that maintains constant speed. In Figure 17, it can be observed the velocity profile applied for the experimentation of frictions.

The algorithm applied for the non-linear control of one of the arms was the following Algorithm 1:
**Algorithm 1** Non-Linear Controller Execution**Result:** Torque signals to be applied by GYEMS motorsEach loop is timed per interruption at 10 ms**while do**    **if** “writing” is equal to 0 **then**        Generate position and velocity profiles        Compute control law and obtain torques in N/m        Scale the torque signals from N/m to Amp        Transform torques to communication CAN BUS signal        Set “writing” to 1    **else**        Request position and speed information by CAN BUS        Perform scaling of position and velocity signals        Apply filter to the speed reference        Set “writing” to 0    **end if**    **end while**

**Figure 18:**
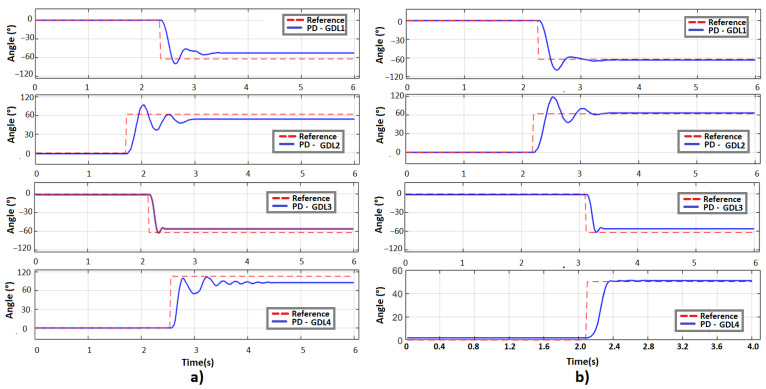
Position regulation for the 4 DOF for left arm: (**a**) only PD; (**b**) PD with gravity compensation.

This algorithm was the same as applied for the Backstepping controller for both the arms and the head. A first experiment was performed using only the PD controller applying gains obtained by the Ziegler-Nichols method for each motor and then with the PD controller with gravity compensator, obtaining the results shown in Figure 18 at step inputs.

**Figure 19:**
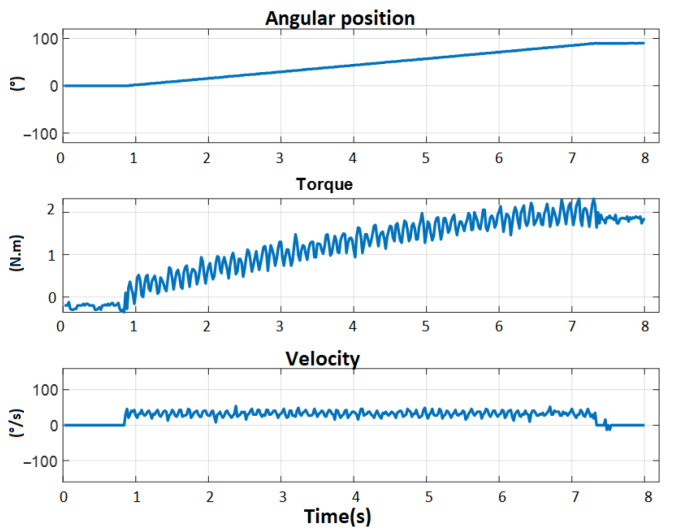
Position regulation with trapezoidal speed profile for the GL1 (motor 1) of the left arm.

Thus, we can see that gravity compensation solves the problem of the error in steady state and in this way, it is possible to apply it in with the generation of trajectories with trapezoidal profiles to obtain the frictions as can be seen in Figure 19.

**Figure 20:**
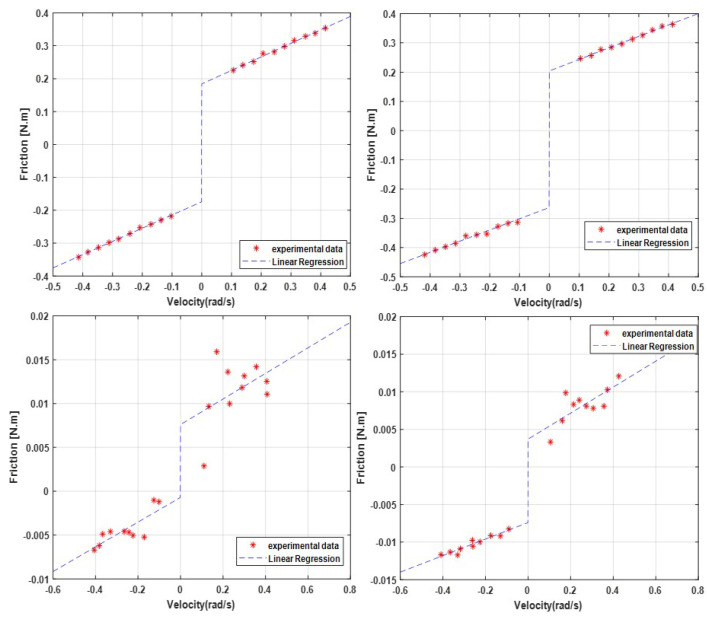
Maps of speed vs. Friction of each motor in left arm.

In this way, we obtain in Figure 20 and Table 5, repeating the same experiment at different speeds and for each motor, the friction maps with the missing coefficients for the mathematical model.

At this point in the experiments, it can be observed that motors 1 and 2 maintain a very similar speed profile and the same for motors 3 and 4. This is because the first two motors are of the same GYEMS RMD motor model -X8 Pro with 6:1 reducer while motors 3 and 4 are GYEMS motors of the RMD L-70 model with lower torque capacity.

### 5.2. Backstepping Control Implementation

**Figure 21:**
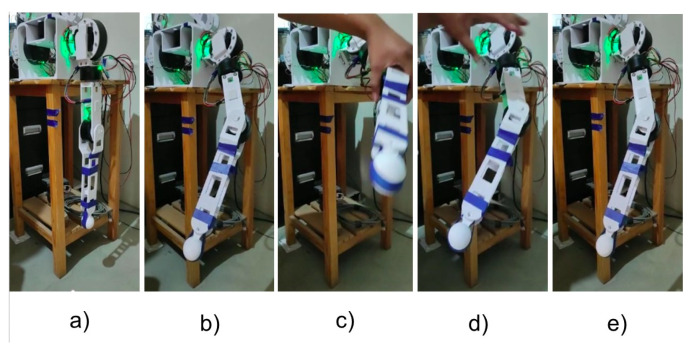
Frame of Left Arm positioned at [ −30${}^{{}^{\circ}}$; 30${}^{{}^{\circ}}$; 60${}^{{}^{\circ}}$; 30${}^{{}^{\circ}}$]. (**a**) ZERO position; (**b**) desired position; (**c**) applying an external disturbance; (**d**) post-disturbance position regulation; (**e**) final position.

Now that we have all the parameters validated for the mathematical model and with the comparison tests performed in the design and simulation stage (according to the discussion in the previous section), backstepping control is implemented. To verify the effectiveness of the implemented control, a first test performed out with changes in angles with a step input and with a disturbance applied to the system. Figure 21 shows the tests carried out applying an external disturbance.

**Figure 22:**
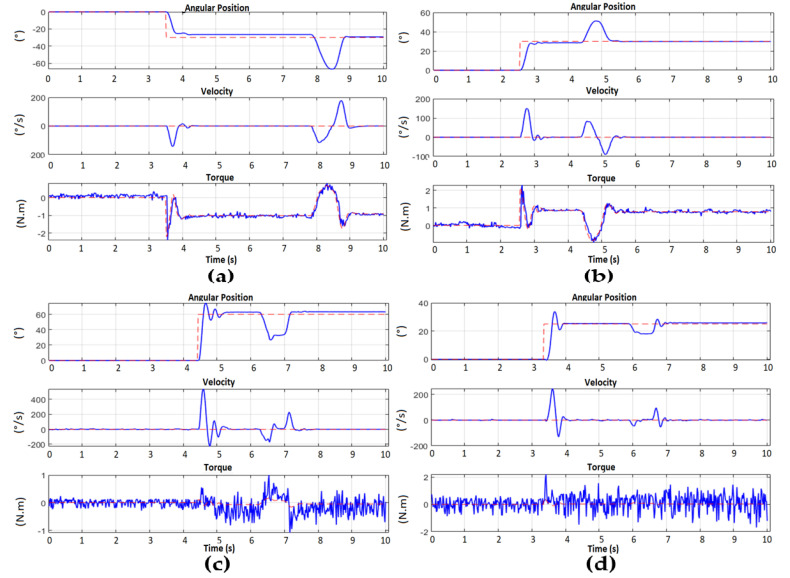
Left arm position regulation with subsequent disturbance. (**a**) Motor 1 from 0${}^{{}^{\circ}}$ to −30${}^{{}^{\circ}}$; (**b**) Motor 2 from 0${}^{{}^{\circ}}$ to 30${}^{{}^{\circ}}$; (**c**) Motor 3 from 0${}^{{}^{\circ}}$ to 60${}^{{}^{\circ}}$; (**d**) Motor 4 from 0${}^{{}^{\circ}}$ to 30${}^{{}^{\circ}}$.

It is observed that the arm reaches the desired position, and seconds later an external force is applied that changes the position and the Backstepping algorithm is responsible, after the disturbance, for repositioning the arm to the requested position. In Figure 22, we can observe the simultaneous position of each motor of the left arm. These data are obtained through UART serial communication to MATLAB from the STM32.

**Figure 23:**
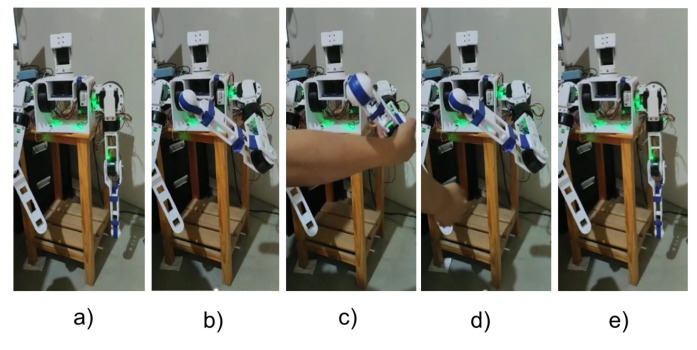
Frame of Left Arm positioned at [ −59${}^{{}^{\circ}}$; 18${}^{{}^{\circ}}$; 61${}^{{}^{\circ}}$; 97${}^{{}^{\circ}}$]. (**a**) ZERO position; (**b**) Desired position; (**c**) Applied disturbance; (**d**) post-disturbance position regulation; (**e**) Final position.

**Figure 24:**
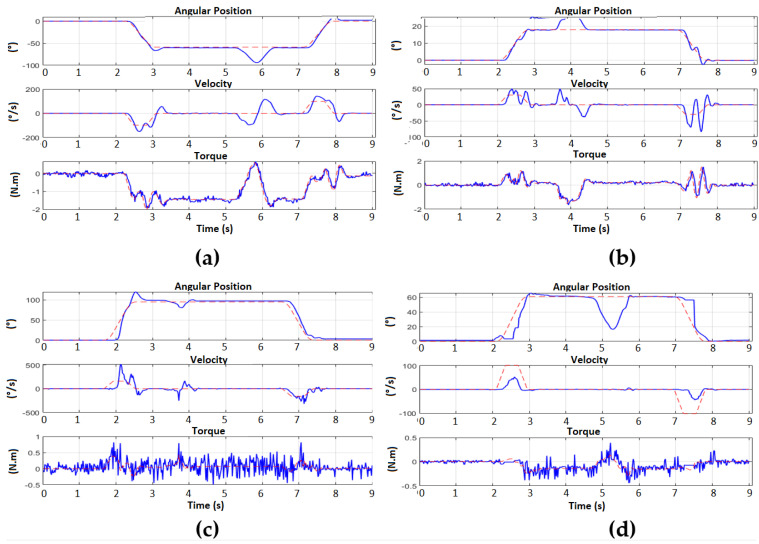
Position and speed regulation with subsequent disturbance. (**a**) Motor 1 from 0${}^{{}^{\circ}}$ to −59${}^{{}^{\circ}}$; (**b**) Motor 2 from 0${}^{{}^{\circ}}$ to 18${}^{{}^{\circ}}$; (**c**) Motor 3 from 0${}^{{}^{\circ}}$ to 61${}^{{}^{\circ}}$; (**d**) Engine 4 from 0${}^{{}^{\circ}}$ to 97${}^{{}^{\circ}}$.

When verifying the effectiveness of the Backstepping control in the regulation of the position, the generation of trajectories with trapezoidal profiles have been applied to take the wrist of the arm to a new different point in the cartesian space and return it to the ZERO position. This second test was performed by applying a disturbance during the resting state at the desired point of the movement. In Figure 23, it is possible to observe the movements executed by the arm. In this way, when applying a trajectory generation, it is observed that it is possible to control the way in which the arm can reach the desired reference, making a more natural movement in terms of speed, because the position and speed reference is being applied at the same time through the controller (see Figure 24).

In the same way as the first experiment, a better control can be observed for motors 1 and 2, which correspond to the motors with gearbox, which present better follow-up of the torque requested by the main Backstepping control, while in the case of motors 3 and 4, which do not have a reducer, the torque presents noise and therefore a more complicated follow-up, but still with regulation of position and speed. The disturbance applied in steady state also allows us to confirm that the regulation for all the motors of the arm is always achieved successfully.

**Figure 25:**
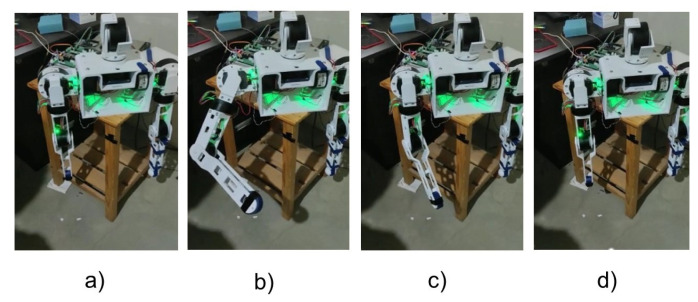
Movement frame of the right arm. (**a**) ZERO position; (**b**) desired position; (**c**) arm return to zero position; (**d**) final position.

**Figure 26:**
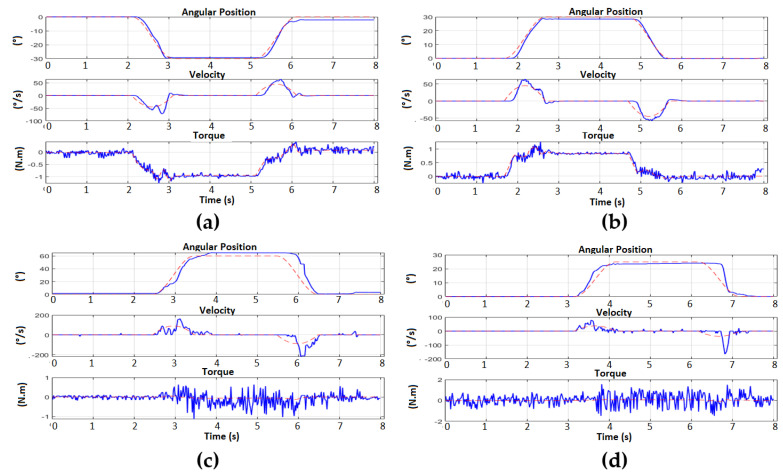
Right arm movements by generating polynomial trajectories. (**a**) Motor 1 from 0${}^{{}^{\circ}}$ to −30${}^{{}^{\circ}}$; (**b**) Motor 2 from 0${}^{{}^{\circ}}$ to 30${}^{{}^{\circ}}$; (**c**) Motor 3 from 0${}^{{}^{\circ}}$ to 60${}^{{}^{\circ}}$; (**d**) Motor 4 from 0${}^{{}^{\circ}}$ to 25${}^{{}^{\circ}}$.

By performing the experiment at another cartesian point, we are verifying a controller expansion in a workspace with a slight change in controller gains. This test is motivated to perform a new experiment for a different point but this time applying softer movements through the use of polynomial trajectories of degree three to the right arm. With this, a smoother movement of the arm could be observed to reach the desired point. Figure 25 and Figure 26 confirm these results because the requested torques are lower than the trapezoidal paths. It has been possible to verify the effectiveness of the Backstepping controller for both arms.

**Figure 27:**
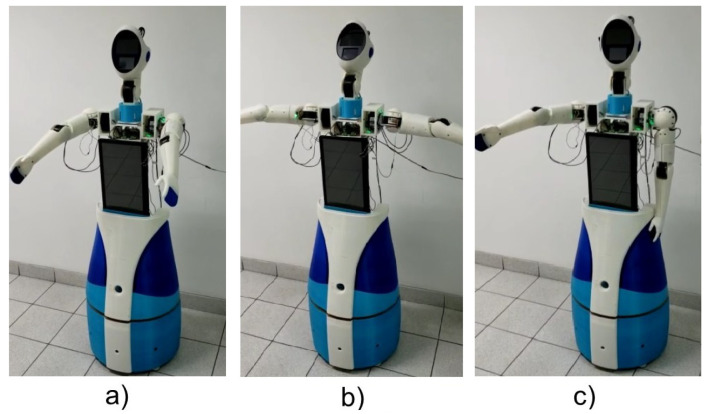
Nod of the robot head. (**a**) ZERO position; (**b**) Desired position [20${}^{{}^{\circ}}$; 30${}^{{}^{\circ}}$]; (**c**) Final position of the movement.

**Figure 28:**
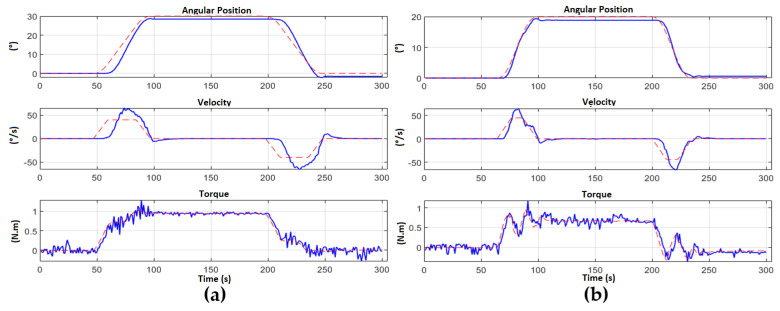
Position, speed and torque of each motor of the teleoperator robot head. (**a**) Motor 1 from 0${}^{{}^{\circ}}$ to 30${}^{{}^{\circ}}$; (**b**) Motor 2 from 0${}^{{}^{\circ}}$ to 20${}^{{}^{\circ}}$.

Additionally, the same control has been tested for the head system. In Figure 27, we can observe the assembled robot with a nod of the head (back and forth) directed by Backstepping control. Finally Figure 28 shows the generation of trapezoidal trajectories.

## 6. Discussion

With the design, implementation, and testing of the proposed control scheme, it has been possible to provide a basis for the technological development of nonlinear controllers for complex multivariable systems. In addition, the analysis of different controllers used in this work provides us with a comparative reference for its implementation to other types of mechanical systems.

As a result of the different experiments performed in the prototype of this project, it has been possible to verify the effectiveness of a purely non-linear controller such as Backstepping compared to a partially adapted linear controller such as PD control with gravity compensation. For this point, in addition to the results already presented, Table 6 shows a comparison of the gains of the controllers for the movements made by the left arm.

In the case of PD control with gravity compensation, it has been possible to observe that a minimum change in the values of the gains causes very different movement responses for the same conditions of the experiment, to the point of leading the system, in some cases, to instability. This further complicates the selection of gains for this controller which must change often depending on the desired motion. This allows us to think of an adaptive solution for the automatic change of these gains and in this way generalize the controller for a workspace.

In the case of the Backstepping control, the selected initial gains have been generalized for all the movements tested in the arm, having to make a minimum adjustment.

In this way, the present research is a starting point for the implementation of adaptive control laws or as a combination of the controllers used here with other controllers based on sliding modes and adaptive control.

The programming of these controllers and the generation of trajectories provide us with the necessary tools to improve the movements performed by the arms and the head of the robot, even currently working on a combination of the generation of trajectories implemented for the formation of movements. composed and brought to a stage where the robot performs a complex presentation of its functions as part of the robot’s social interaction routine with people.

## 7. Materials and Methods

The presented work performs the implemented tests of a Backstepping control for the arms and head of a teleoperated robot. The details of the movements made during the tests with the initial prototype are available to any researcher at the link specified in [7].

## 8. Conclusions

A control scheme for the tracking of trajectories and a profile of speeds that execute the movements of the arms and head of a robot with natural motion similar to people was presented. Thus, it was possible to demonstrate the effectiveness both in simulation and experimentation in real time. The control strategy used is based on the information provided by an encoder of the position and speed of each motor with internal torque regulation with PI control through the CAN BUS protocol to the central Backstepping control that ensures the positioning of the motors for both arms and head of the robot. The work presented also made a comparison of the main nonlinear controllers on which it has been possible to distinguish the Backstepping control as the most appropriate regulator for the implementation presented, as well as some additional features as a result of this comparison. During the development of the non-linear controller algorithm, a procedure was performed that allows real-time communication of up to four (04) GYEMS motors with a single STM32 microcontroller and additional simultaneous communication of three (03) STM32 microcontrollers with a central navigation system through serial protocols. The results obtained for each movement were satisfactory since the control objectives were achieved, obtaining a precise control system that allows the freedom to regulate the way in which the robot arms and head reach the points that the robot routine requires.

As future work, the Backsteeping control will be implemented to execute several routines, for which an automated calibration process will be developed to reduce the required time to optimize the parameters of the controller for different routines. Additionally, variations of the speed of the arms will be evaluated during the interaction with survey participants to determine the perception of the robot gestures based on their speed.

## Figures and Tables

**Table 1:** Requirements for movable limbs design.

Design Requirements	
Total weight	5 kg
Torque at shoulder	2.1 Nm (Flexion, extension)
	2.1 Nm (Abduction)
	0.36 Nm (Internal/external rotation)
Torque at elbow	0.36 Nm (Flexion)
Joint speed range	≈50–150 deg/s
System Autonomy	1 h
Human dimensions	≈33.3 cm shoulder—elbow
(proportional to a	≈29 cm elbow—knuckles
1.65 m female)	≈7.3 cm shoulder—chin

**Table 2:** Values of Finite element analysis on Autodesk Inventor of 3D printed arm pieces.

	Von Mises Stress (MPa)		Displacement (mm)		Security Factor	
	**Min**	**Max**	**Min**	**Max**	**Min**	**Max**
Shoulder 1	0	33.6	0	0.1595	6.15	15
Shoulder 2	0	37.64	0	0.05455	7.74	15
Arm	0	23.81	0	0.1296	8.69	15
Forearm	0	19.4	0	0.1801	10.67	15

**Table 3:** Technical information of the prototype of the robot arms and head.

Technical Information	
Controllers	STM32F446ZE x3
Communication	CAN, UART
Head output	Image, audio
Power	8.5 W
Motor operation Voltage	24 V
Control operation Voltage	5 V
Current consumption	1.2 A
System Autonomy	1 h

**Table 4:** Comparison of nonlinear controllers against polynomial trajectories.

	PD + G	Backstepping	SMC
Circular path error	15.93%	3.63%	3.23%
Path error polynomial	8.0307%	2.1541%	2.0206%
Execution time	6.7 s	7.6 s	10.8 s
Disturbance control	average	High	High
Setpoint changes	average	High	High
Feedback variables	Pos,vel	Pos,vel	Pos,vel,Acc
Smooth actuator changes	Yes	Yes	No

**Table 5:** Table of friction coefficients for each motor on the left arm.

V+/V−		V+/V−	
**an**	**an**−	**bn**	**bn**−
0.4106	0.4021	0.1842	−0.1742
0.3924	0.3834	0.2044	−0.3631
0.0146	0.0141	0.0076	−0.0007
0.0173	0.011	0.0037	−0.0074

**Table 6:** Gain values for the nonlinear controllers in different movements of the left arm.

	PD Control with Gravity			Backstepping Control		
	**Step**	**Spline**	**Polinomial**	**Step**	**Spline**	**Polinomial**
Kp11	5.2	9.6	3.2	15	15	15
Kp22	5.1	10.3	3.4	15	17	15
Kp33	0.5	0.87	0.22	120	118	120
Kp44	0.35	0.95	0.19	120	120	120
Kv11	1.1	2.2	0.6	6	13	8
Kv22	1.2	0.19	0.4	6	10	4
Kv33	0.04	0.07	0.05	6	4	2
Kv44	0.06	0.09	0.06	5	6	4

## Data Availability

The data presented in this study is available on request from the corresponding author.
